# Tetraspanin proteins in membrane remodeling processes

**DOI:** 10.1242/jcs.261532

**Published:** 2024-07-25

**Authors:** Raviv Dharan, Raya Sorkin

**Affiliations:** 1 School of Chemistry, Raymond & Beverly Sackler Faculty of Exact Sciences, Tel Aviv University, 6997801, Tel Aviv, Israel; 2 Center for Physics and Chemistry of Living Systems, Tel Aviv University, 6997801, Tel Aviv, Israel

**Keywords:** Tetraspanin, Membrane biophysics, Membrane remodeling, Membrane dynamics, Membrane curvature, Tetraspanin-enriched microdomains

## Abstract

Membrane remodeling is a fundamental cellular process that is crucial for physiological functions such as signaling, membrane fusion and cell migration. Tetraspanins (TSPANs) are transmembrane proteins of central importance to membrane remodeling events. During these events, TSPANs are known to interact with themselves and other proteins and lipids; however, their mechanism of action in controlling membrane dynamics is not fully understood. Since these proteins span the membrane, membrane properties such as rigidity, curvature and tension can influence their behavior. In this Review, we summarize recent studies that explore the roles of TSPANs in membrane remodeling processes and highlight the unique structural features of TSPANs that mediate their interactions and localization. Further, we emphasize the influence of membrane curvature on TSPAN distribution and membrane domain formation and describe how these behaviors affect cellular functions. This Review provides a comprehensive perspective on the multifaceted function of TSPANs in membrane remodeling processes and can help readers to understand the intricate molecular mechanisms that govern cellular membrane dynamics.

## Introduction

Tetraspanins (TSPANs) constitute a family of transmembrane proteins present in almost every cell type, with 33 known members in humans. TSPANs have been associated with key cellular and pathological functions such as cell adhesion (Zuidema et al., 2020), immune signaling (Levy and Shoham, 2005b), cell–cell fusion (Le Naour et al., 2000; Miyado et al., 2000), viral infection (Hantak et al., 2019) and cancer metastasis (Vences-Catalán et al., 2021). Membrane remodeling underlies these events and is associated with proteins from the TSPAN family. Interactions of TSPANs with one another, as well as with other membrane proteins, are integral to their mode of action and are thought to play a major role in membrane compartmentalization by forming TSPAN-enriched microdomains (TEMs) (Hemler, 2005; Le Naour et al., 2006; Charrin et al., 2009; Yáñez-Mó et al., 2009; Charrin et al., 2014; Zuidscherwoude et al., 2015; van Deventer et al., 2021). In these microdomains, which are also referred to as ‘the TSPAN web’ (Boucheix and Rubinstein, 2001; Levy and Shoham, 2005a), TSPANs are considered to be molecular organizers that can recruit associated proteins to exert their cellular functions (Earnest et al., 2017). TSPANs have been shown to have different levels of interactions (Charrin et al., 2003a); they can interact directly with proteins such as integrins (Yauch et al., 1998; Serru et al., 1999) and lipids such as cholesterol (Charrin et al., 2003b). Additionally, TSPANs can interact indirectly with other proteins and lipids in the web (Yáñez-Mó et al., 2009; Charrin et al., 2014); for example, the TSPANs CD9, CD81, CD82 and CD151 can interact with CD46 via integrins (Lozahic et al., 2000), and CD81 can interact with CD21 (also known as CR2) via CD19 (Matsumoto et al., 1993). The TEM model, however, has recently been revisited by studies showing that several TSPANs can also assemble into smaller-scale clusters termed nanoclusters (Zuidscherwoude et al., 2015). In particular, it has been demonstrated that TSPANs are organized in nanodomains that can be in close proximity but not in the same domain (Zuidscherwoude et al., 2015; Dahmane et al., 2019).

**Fig. 1:**
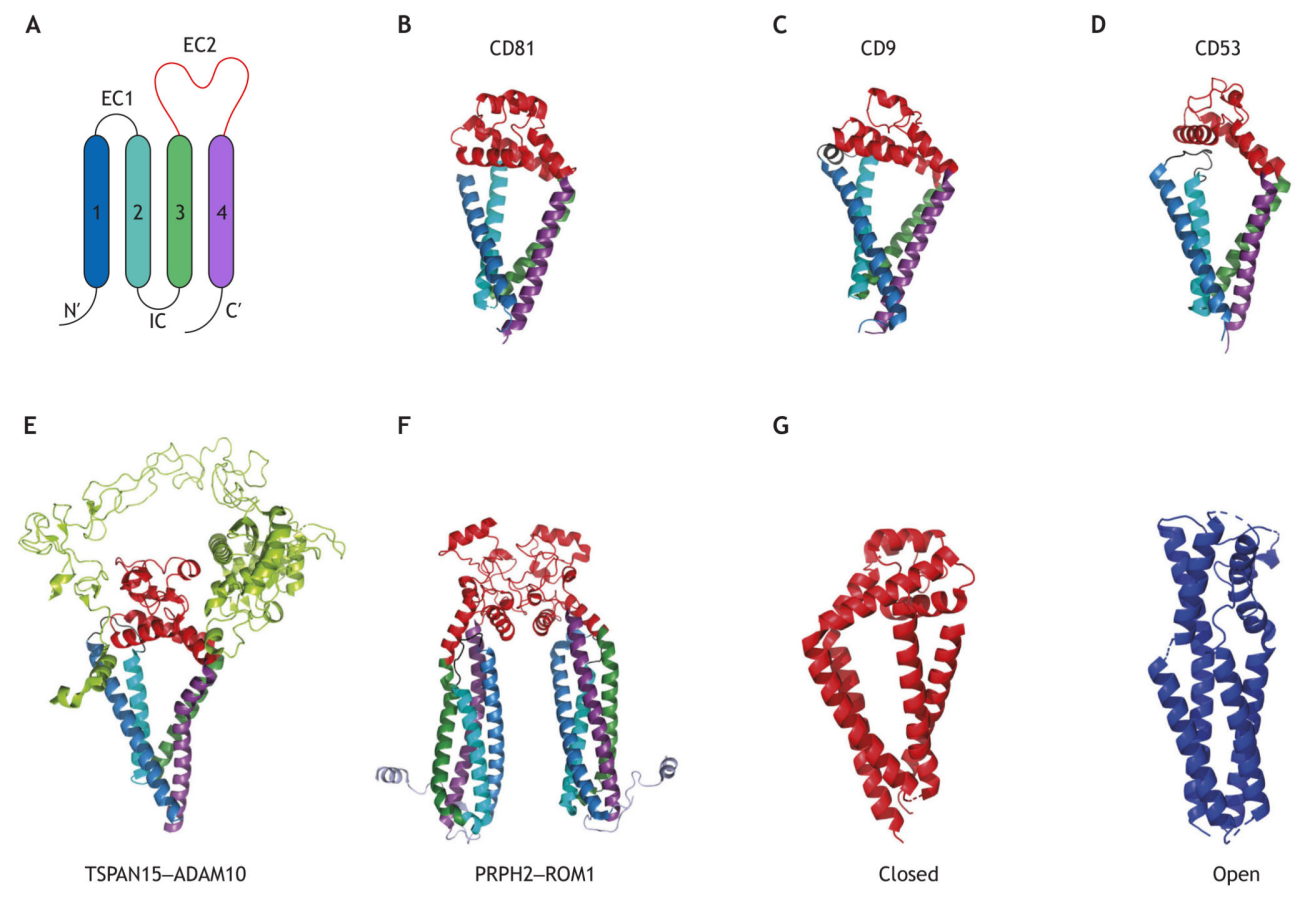
Structures of TSPANs. (A) Schematic representation of the general TSPAN structure. The schematic illustrates the N and C termini; the extracellular loop EC1, connecting TM1 and TM2; the short intracellular loop (IC), connecting TM2 and TM3; and the extracellular loop EC2, connecting TM3 and TM4. (B–D) Crystal structures of (B) CD81 (PDB 5TCX; Zimmerman et al., 2016), (C) CD9 (PDB 6K4J; Umeda et al., 2020) and (D) CD53 (PDB 6WVG; Yang et al., 2020). (E) Cryo-EM structure of the TSPAN15–ADAM10 complex (PDB 8ESV; Lipper et al., 2023). (F) Cryo-EM structure of a PRPH2–ROM1 heterodimer (PDB 7ZW1; El Mazouni and Gros, 2022). In panels A–F, the main protein domains are colored as follows: TM1, blue; TM2, cyan; TM3, dark green; TM4, purple; EC1, black; EC2, red. ADAM10 is shown in light green. (G) Closed (PDB 5TCX; Zimmerman et al., 2016) and open (PDB 7JIC; Susa et al., 2021) conformations of CD81. Structures generated with PyMOL.

Recent evidence suggests that the mechanism of action of TSPANs in biological systems is tightly linked to membrane tension and curvature. Structural studies show that several TSPANs have an inverted cone shape (Fig. 1) (Zimmerman et al., 2016; Umeda et al., 2020; Yang et al., 2020; Lipper et al., 2023). Furthermore, a link between TSPAN enrichment and curved membrane morphology has been demonstrated; for example, TSPANs CD9 and CD81 are enriched in the growing tips of virus buds (Dahmane et al., 2019) and in egg cell microvilli (Kaji et al., 2000; Runge et al., 2007), where they play a vital role in egg–sperm fusion (Le Naour et al., 2000; Miyado et al., 2000; Rubinstein et al., 2006; Jégou et al., 2011). Likewise, TSPANs CD81 and CD82 are found in micro-protrusions of various cell types (Bari et al., 2011; Zhang and Huang, 2012). Furthermore, several TSPANs preferentially partition from low-curvature membranes into highly curved membrane tethers (Dharan et al., 2022), explaining their localization in curved cellular membranes. TSPANs are also essential for the formation of migrasomes, the transient organelles that form on cellular retraction fibers. These fibers are the cylindrical protrusions of cell membranes that emerge during cell migration along external substrates (Huang et al., 2019; Zhang et al., 2020; Yu and Yu, 2022). Migrasomes have an important role in mediating communication between cells, among other emerging functions, as we elaborate on later in the text.

Here, we review recent findings on the involvement of TSPANs in membrane remodeling events, including their structure and distinct structural features, as well as evidence that demonstrates curvature-directed localization of TSPAN proteins. We address the tendency of TSPANs to form membrane domains and the involvement of such domains in cellular processes like membrane fusion, damage repair, cancerous tumor development and migrasome formation. Finally, we review methods for characterization of TSPAN organization and function. We conclude with an outlook discussing future research directions aimed at elucidating the mechanisms of action of TSPANs and, in particular, the biophysical aspects that should be addressed in future studies. As TSPANs are directly involved in multiple cellular processes, understanding their function will have far-reaching implications in medicine and biology.

## Structure of TSPANs

TSPANs are small transmembrane proteins, ranging from 22 to 39 kDa, with four transmembrane helices (TM1 to TM4) linked by extracellular loops (EC1 and EC2) and an intracellular loop (Fig. 1A). EC2 is a relatively large and variable protein loop and is associated with various TSPAN interactions; it has been shown that the EC2 of CD81 can assume different configurations (Kitadokoro et al., 2002; Susa et al., 2020). The plasticity within the EC2 might allow CD81 to dynamically reorient to interact with structurally different partners (Susa et al., 2023).

TSPANs also contain short N-terminal and C-terminal cytoplasmic tails, which vary between different TSPANs (Fig. 1A). These cytoplasmic tails can interact with cellular proteins like the adaptor protein complex AP3 (Rous et al., 2002), protein kinase C (Zuidscherwoude et al., 2017), and Rac GTPases (Tejera et al., 2013), as well as direct intracellular signals for endocytic trafficking (Duffield et al., 2003; Berditchevski and Odintsova, 2007; Van Deventer et al., 2017). All TSPANs have intracellular and membrane-adjacent regions that include cysteine palmitoylation sites (Yáñez-Mó et al., 2009). On the outer membrane side, EC2 contains a conserved Cys-Cys-Gly (CCG) motif and cysteine residues that are linked by 2–4 disulfide bonds (Hemler, 2008; Susa et al., 2023). The disulfide bonds are crucial for proper folding of the ectodomain, and TSPANs can be classified according to the number of these bonds (Susa et al., 2023). Palmitoylated cysteine residues are thought to be essential for efficient interactions between TSPANs and other associated proteins (Stipp et al., 2003; Levy and Shoham, 2005b). TSPAN–TSPAN interactions are also regulated by cholesterol and gangliosides (glycosphingolipids found in the outer leaflet of the plasma membrane) (Charrin et al., 2003b; Odintsova et al., 2006; Silvie et al., 2006). In fact, it has recently been demonstrated that gangliosides affect the diffusion of certain TSPANs within the plasma membrane (Fernandez et al., 2021).

In contrast to the more variable EC2 loop, the transmembrane region of TSPANs is highly conserved and has an inverted cone shape in several proteins, as exemplified by the crystal structure of full-length CD81 (Zimmerman et al., 2016) (Fig. 1B). CD81 has an inverted cone-like shape, in which the transmembrane segments pack as two largely separate pairs of helices that are capped by the EC2 loop at the outer membrane leaflet and converge at the inner membrane leaflet. This architecture creates an intramembrane hydrophobic pocket, which is proposed to be a cholesterol-binding site (Zimmerman et al., 2016). The crystal structure of CD9 (Fig. 1C) has also been shown to have the same inverted cone shape and has been proposed to generate membrane curvature, accounting for the localization of CD9 in curved membranes such as the microvilli of oocytes (Umeda et al., 2020). The cryo-electron microscopy (cryo-EM) structure of CD9 in complex with its partner protein EWI-2 [also known as IGSF8; a member of the immunoglobulin subfamily characterized by the presence of an extracellular Glu-Trp-Ile (EWI) motif] further demonstrates that the outer side of TM3 and the EC2 loop are important for TSPAN interactions with other proteins (Umeda et al., 2020). The structural arrangement of CD9–EWI-2 is similar to that of the complex formed by CD9 and EWI-F (also known as PTGFRN) (Oosterheert et al., 2020), in line with the partially overlapping reported functions of the partner proteins (Stipp et al., 2001; Sala-Valdés et al., 2006). Furthermore, CD53 and TSPAN15 (Fig. 1D,E) have the same inverted cone shape (Yang et al., 2020; Lipper et al., 2023).

Two distinct TSPAN conformations, open and closed, involving a conformational change in EC2 orientation (Fig. 1G) have been suggested; molecular dynamics simulations of CD81 suggest that the transition between open and closed conformations of this protein is dependent on cholesterol binding, which promotes the closed conformation (Zimmerman et al., 2016). By contrast, molecular dynamics simulations of CD9 suggest that this transition occurs spontaneously even in the absence of cholesterol (Umeda et al., 2020), whereas for CD53, the open conformation is supported by EC1–EC2 interactions and is required for interactions between TSPANs and partner proteins (Yang et al., 2020).

A closed TSPAN conformation has also been observed in the TSPAN15–ADAM10 complex (Lipper et al., 2023) (Fig. 1E). In this complex, EC2 is located over the opening formed by the transmembrane helices of TSPAN15, similar to its orientation in reported isolated TSPAN structures of CD81, CD9 and CD53 (Zimmerman et al., 2016; Umeda et al., 2020; Yang et al., 2020). The EC2 conformation of CD81 is different when it interacts with the immunoglobulin single-pass transmembrane protein CD19 to form the CD81–CD19 complex, which is crucial for proper B cell function (Susa et al., 2021). In the CD81–CD19 complex, the EC2 loop pivots ~60° relative to the membrane plane, while the transmembrane helices draw closer together, eliminating the central cavity and thus likely blocking cholesterol binding. Furthermore, other TSPANs, like peripherin-2 (PRPH2) and rod outer segment protein 1 (ROM1), have a less-conical structure compared to those of other TSPANs (Fig. 1F), yet PRPH2–ROM1 heterodimers and higher-order oligomers do form curved structures (El Mazouni and Gros, 2022). Other TSPAN proteins also deviate from the inverted cone-like shape; based on their cryo-EM structure, the TSPAN proteins uroplakin-1a and uroplakin-1b are suggested to have a rod-like shape in the uroplakin array structure (Min et al., 2006). The structural differences between TSPANs, which are mainly in the EC2 loop, might modulate different interactions with their associated proteins. Nevertheless, the inverted cone shape of several TSPANs (or their oligomers), indicates that the physical properties of the membrane, such as membrane curvature, can influence or be influenced by these proteins.

## TSPANs sense and generate membrane curvature

**Fig. 2:**
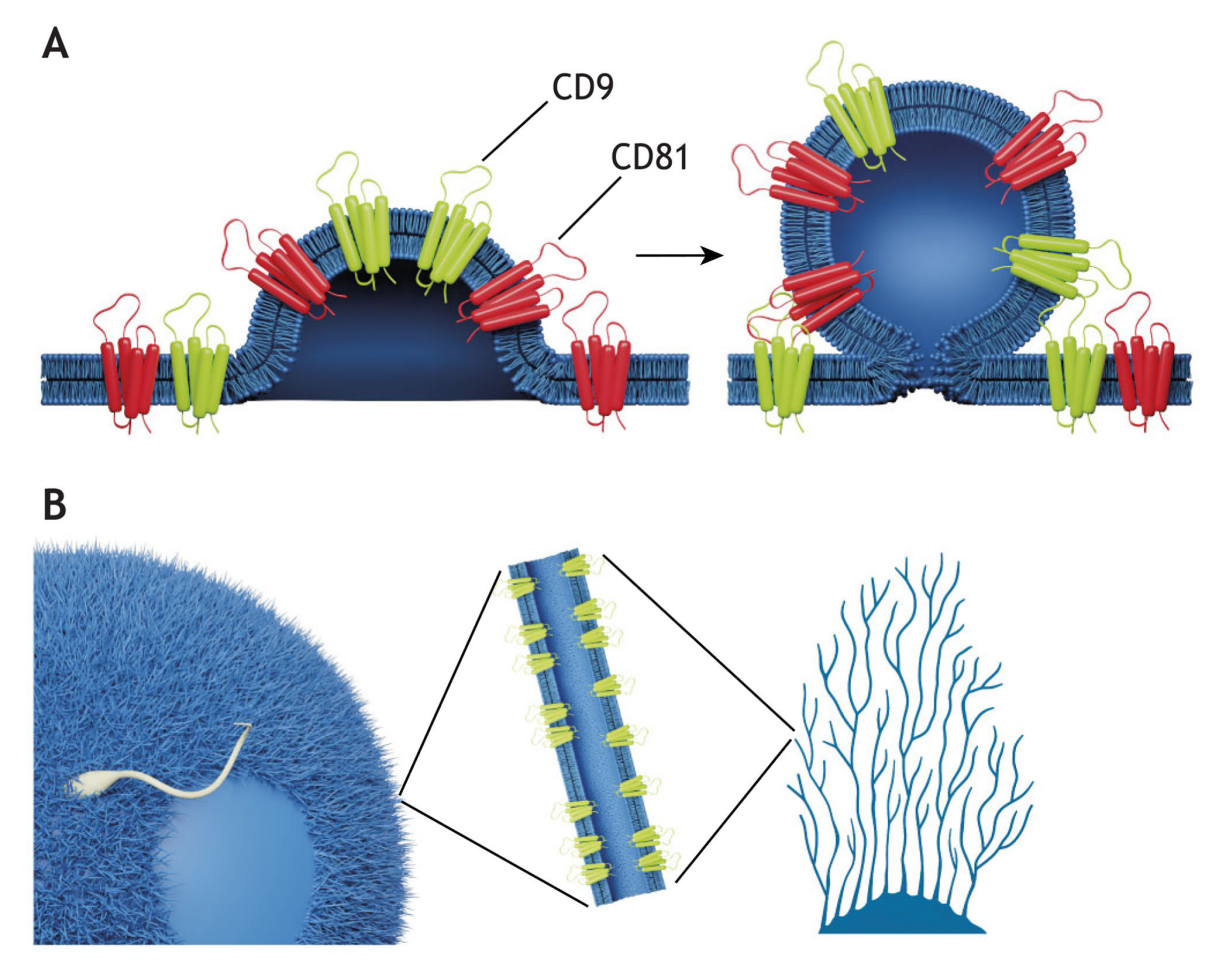
TSPANs are enriched in curved membranes. (A) Schematic illustration showing TSPAN enrichment (CD9 and CD81 depicted as examples) in exosomes during exosome formation, driven by high membrane curvature. (B) Schematic illustration of an egg cell with microvilli and an attached sperm cell (left), and retraction fibers generated at the rear of a migrating cell (right). The image shown in the middle depicts a cross section of a curved membrane tube, highlighting that both microvilli and retraction fibers are membrane tubes exhibiting high curvature, which induces TSPAN enrichment.

Several studies have demonstrated that TSPAN proteins are localized in curved membranes (Fig. 2). For example, CD9, CD81, CD63 and CD82 are markers of extracellular vesicles (EVs) (Willms et al., 2018; Presle et al., 2021; Rai et al., 2021). TSPANs are one of the most prevalent types of protein in the membranes of EVs, particularly in exosomes (Fig. 2A), which have high membrane curvature due to their small size (Rana and Zöller, 2011). Furthermore, multiple studies have demonstrated that TSPANs are not merely markers for EVs; TSPANs are involved in various aspects of EVs, including their formation (van Niel et al., 2011) (Chairoungdua et al., 2010), interactions with partner proteins (Castro-Cruz et al., 2023), cargo selectivity (Andreu and Yáñez-Mó, 2014), cell targeting (Rana et al., 2012) and fusion (Jankovičová et al., 2020). However, recent studies have challenged the significance of TSPANs in cargo selectivity of EVs, demonstrating that CD6, CD9 and CD81 might have less importance in this function than previously thought (Fan et al., 2023; Tognoli et al., 2023).

CD9, CD81 and CD63 are also enriched in the HIV-1 viral envelope as a result of their localization in Gag-induced virus budding sites (Nydegger et al., 2006; Dahmane et al., 2019). CD9 and CD81 are also enriched in egg cell microvilli (Fig. 2B), and it has been demonstrated that in the absence of CD9, the shape of the microvilli becomes abnormal (Runge et al., 2007). Additionally, TSPANs are localized in cell protrusions and regulate their formation and development (Israels and McMillan-Ward, 2007; Bari et al., 2011; Zhang and Huang, 2012). For example, CD81 overexpression promotes the formation and extension of microvilli, whereas overexpression of CD82 has the opposite effect, leading to a concave cell periphery (Bari et al., 2011). Other TSPANs, like TSPAN4, are abundant in retraction fibers (Fig. 2B). Additionally, TSPANs are localized in the curved membrane structures associated with ‘adhesion zippers’, regions where adjacent cells create tightly interconnected formations along their points of contact. This is exemplified by CD151, which maintains vascular stability in endothelial cells by enhancing cell adhesion zipper formation and managing cytoskeletal tension (Zhang et al., 2014). Likewise, CD9 clusters facilitate the zippering of microvilli between cells and regulate cell–cell fusion (Singethan et al., 2008).

The curvature sensitivity of TSPAN4 and CD9 was directly demonstrated in a recent study where these proteins were enriched by positive membrane curvature (curvature is conventionally defined as positive for convex membrane regions and negative for concave membrane regions). This enrichment was observed within membrane tethers extracted from giant plasma membrane vesicles (Dharan et al., 2022). In this study, using a thermodynamic model for protein partitioning between a flat reservoir and a curved tether, the intrinsic curvatures of the proteins were estimated. The obtained high values of these intrinsic curvatures (0.11 and 0.12 nm^−1^ for TSPAN4 and CD9, respectively) are in line with their inverted cone structures and the observations of TSPANs in positively curved cellular membranes. In a related study, it was suggested that CD82 can regulate membrane tension and affect the shape of small membrane vesicles that are found within migrasomes (Ordas et al., 2021). In this work, membrane tubes were pulled from cells in the presence of CD82, and the tether force (the force that is needed to hold the tether) was measured, where higher forces were attributed to higher membrane tension. However, the bending rigidity of the membrane can also affect the measured pulling force of the tethers. It was suggested that the influence of CD82 on membrane tension might be through an indirect mechanism via caveolin and activation of the transcription factor yes-associated protein (YAP1) (Ordas et al., 2021).

In contrast to the documented positive curvature sensitivity of TSPAN4 and CD9 (Dharan et al., 2022), the TSPANs PRPH2 and ROM1 are able to sense negative membrane curvature. These proteins assemble into structures that impose an extreme radius of curvature at the rims of stacked membrane discs in photoreceptor cells that enable vision (Pöge et al., 2022). In these structures, the luminal protein domains of the TSPAN complexes are important for stabilization of the disc rim scaffold. Mutations in these protein domains might hinder disulfide bond-stabilized oligomerization and consequently impair disc morphogenesis, compromising the structural integrity of rod outer segments and potentially leading to blindness (Pöge et al., 2022). Interestingly, PRPH2–ROM1 oligomers have been found to form structures with positive as well as negative curvatures (Kevany et al., 2013; Milstein et al., 2017; 2020; El Mazouni and Gros, 2022).

Although PRPH2–ROM1 is the only reported case of negative curvature sensitivity by TSPANs, the localization of these proteins in membranes with both negative and positive curvature demonstrates their diverse functionality in cellular processes. Since the transmembrane protein domains are highly conserved among TSPANs, the differences in the curvature affinity of these proteins likely originate from their extracellular loops, specifically the EC2 loop, which varies significantly among TSPANs. The EC2 loop has recently been demonstrated to have crucial function in TSPAN4 membrane curvature sensitivity (Dharan et al., 2024). Deletion of the EC2 loop leads to a significant decrease in TSPAN4 sorting in membrane tethers. Furthermore, the EC2 loop, specifically the small loop within EC2 containing amino acids 151–187, has been suggested to be important for TSPAN4 curvature-induced interactions, as deletion of this loop abolishes TSPAN4 sorting hysteresis (i.e. the sorting values obtained upon tension increase do not match the values upon tension decrease). Therefore, the EC2 loop might affect the extent of curvature sensitivity of the protein along with modulation of its molecular interactions.

## TSPAN-enriched microdomains

The functionality of TSPANs is thought to be dependent on their ability to associate among themselves and with other integral proteins and adhesion molecules, forming a distinct class of membrane domains (TEMs) (Yáñez-Mó et al., 2009; Charrin et al., 2009; Huang and Yu, 2022). Such domains can form in both flat and curved membranes under different circumstances. Below we focus on TEM formation in curved cellular structures.

TEMs are observed along retraction fibers, where TSPANs promote the formation of migrasomes (discussed in more detail below). The formation of TEMs along thin tubular structures such as the retraction fibers indicates that clustering of these TSPANs is likely enhanced due to their curvature-induced enrichment. Moreover, following the enrichment of TSPAN4 in curved membranes, its curvature sensitivity exhibits hysteresis between the path of curvature increase versus curvature decrease. This hysteresis in curvature sensitivity is abolished upon removal of the extracellular part of the protein, which is known to facilitate protein–protein interactions (Dharan et al., 2022). Thus, it appears that curvature induces sorting and protein enrichment, which in turn results in the formation of membrane domains that would not have formed in the absence of sufficiently high concentrations of TSPAN proteins. More research is needed to confirm this idea; for example, single-particle tracking or fluorescence correlation spectroscopy experiments can be used to test whether larger-scale domains form as a result of TSPAN enrichment within highly curved structures.

## TSPANs in migrasome formation

**Fig. 3:**
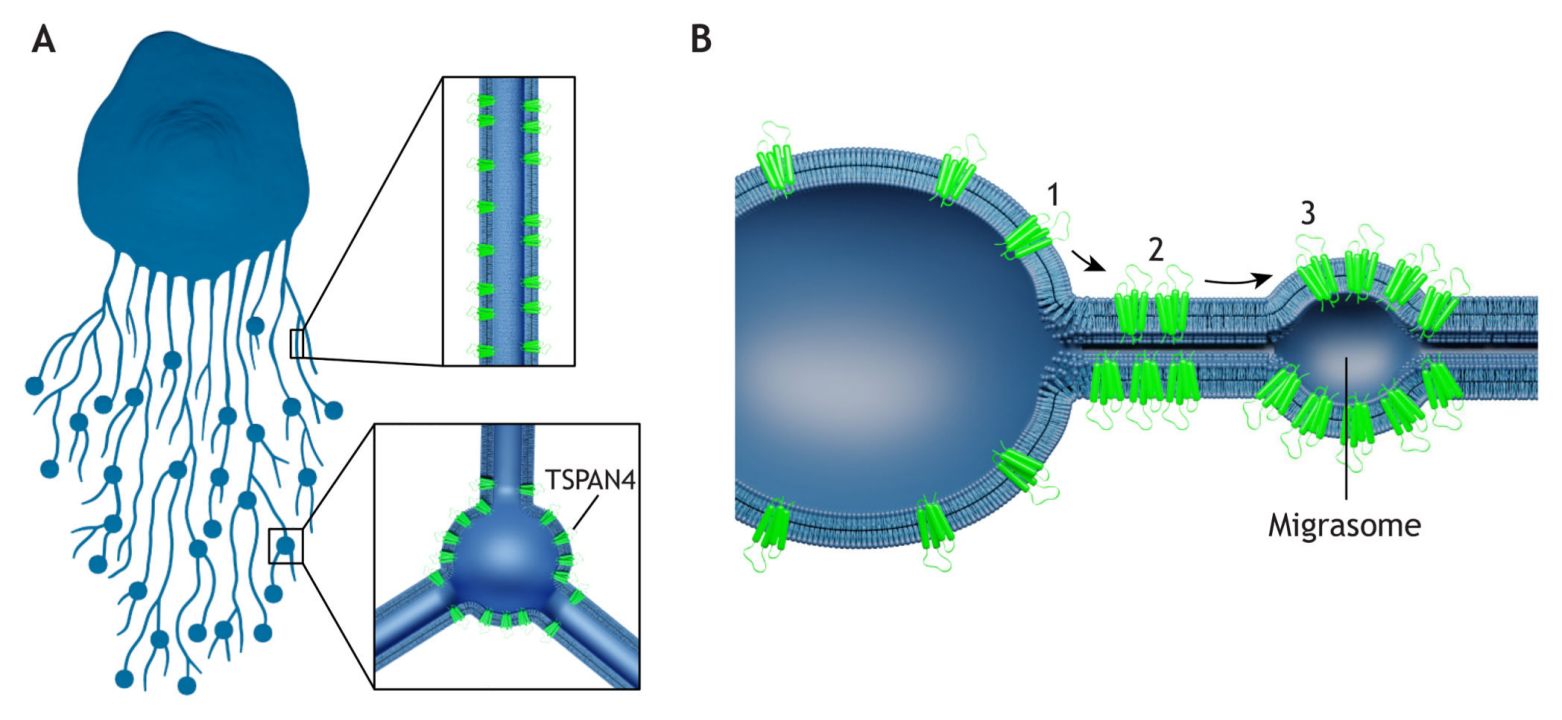
TSPANs are enriched in migrasomes and retraction fibers. (A) Schematic representation of a cell with retraction fibers and migrasomes. The insets show a cross section of a retraction fiber (top) and a migrasome at a three-tube junction (bottom), illustrating that the retraction fibers and migrasomes are enriched with several TSPANs, such as TSPAN4. (B) TSPAN migration during migrasome formation. The illustration depicts a cross section of a retraction fiber with a migrasome pulled from a cell. (1) First, TSPAN4 partitions from the cell to the higher curvature of the retraction fiber. (2) Next, TSPAN4 forms membrane domains in the retraction fiber. (3) Then, TSPAN4 membrane domains become enriched in the migrasome.

TSPANs have been found to be indispensable for the formation of recently discovered cellular organelles called migrasomes (Ma et al., 2015). Migrasomes are formed by the local swelling of retraction fibers during cell migration. They are usually several micrometers in diameter and facilitate the release of cellular contents at specific locations (Fig. 3A). Several studies have shown that migrasomes have important physiological functions *in vivo*. For example, during zebrafish gastrulation, migrasomes act as signaling organelles that provide regional biochemical cues to allow correct cell positioning (Jiang et al., 2019). Additionally, monocytes can deposit migrasomes enriched with pro-angiogenic factors to promote angiogenesis in chick embryos (Zhang et al., 2022). Migrasomes also play a role in regulating mitochondrial quality by accumulating damaged mitochondria and facilitating their removal from migrating cells (Jiao et al., 2021). Furthermore, migrasomes carry mRNA and proteins that can be transferred to recipient cells, thereby modifying their functionality (Zhu et al., 2021). The emerging cellular functions of migrasomes indicate their role as important organelles that are capable of transforming into EVs upon detachment from retraction fibers.

TSPANs play an important role in migrasome formation. Several TSPANs, such as TSPAN4, are migrasome markers (Huang and Yu, 2022; Wu et al., 2017), and overexpression of 14 out of the 33 known mammalian TSPANs enhances migrasome formation (Huang et al., 2019). The presence of TSPANs and cholesterol contributes to the creation of TEMs that mediate migrasome formation (Huang et al., 2019; Huang and Yu, 2022). Recent data demonstrate that changes in membrane tension and curvature lead to formation of migrasome-like vesicles in the presence of TSPAN4 (Dharan et al., 2023). In the first stage of migrasome formation, which is mediated by membrane tension, localized swellings devoid of TSPAN4 appear on the retraction fibers. In the second stage, TSPAN4 proteins migrate towards and onto these swellings, facilitating their growth into migrasomes (Fig. 3B). The recruitment of TSPAN4 to these swellings is essential for the subsequent growth and stabilization of migrasomes. Since TSPANs are first enriched in the curved membrane of retraction fibers and then clustered into TEMs that migrate to the lower membrane curvature of the migrasomes, it has been suggested that individual TSPANs and TEMs have different curvature sensitivities (Dharan et al., 2023). It has been observed that TSPANs can stiffen membranes *in vitro*, and this might also contribute to the migration of TEMs to the migrasomes, as TSPAN membrane domains with high bending rigidity prefer lower-curvature membranes (Huang et al., 2019). Overall, TSPANs serve as essential components in the formation of both retraction fibers and migrasomes. Retraction fibers exhibit a curved membrane structure, which contrasts with the flat morphology of migrasomes. The enrichment of TSPAN4 in both curved and flat membranes underscores its ability to function in diverse membrane geometries.

## TEMs in membrane fusion and damage repair

**Fig. 4:**
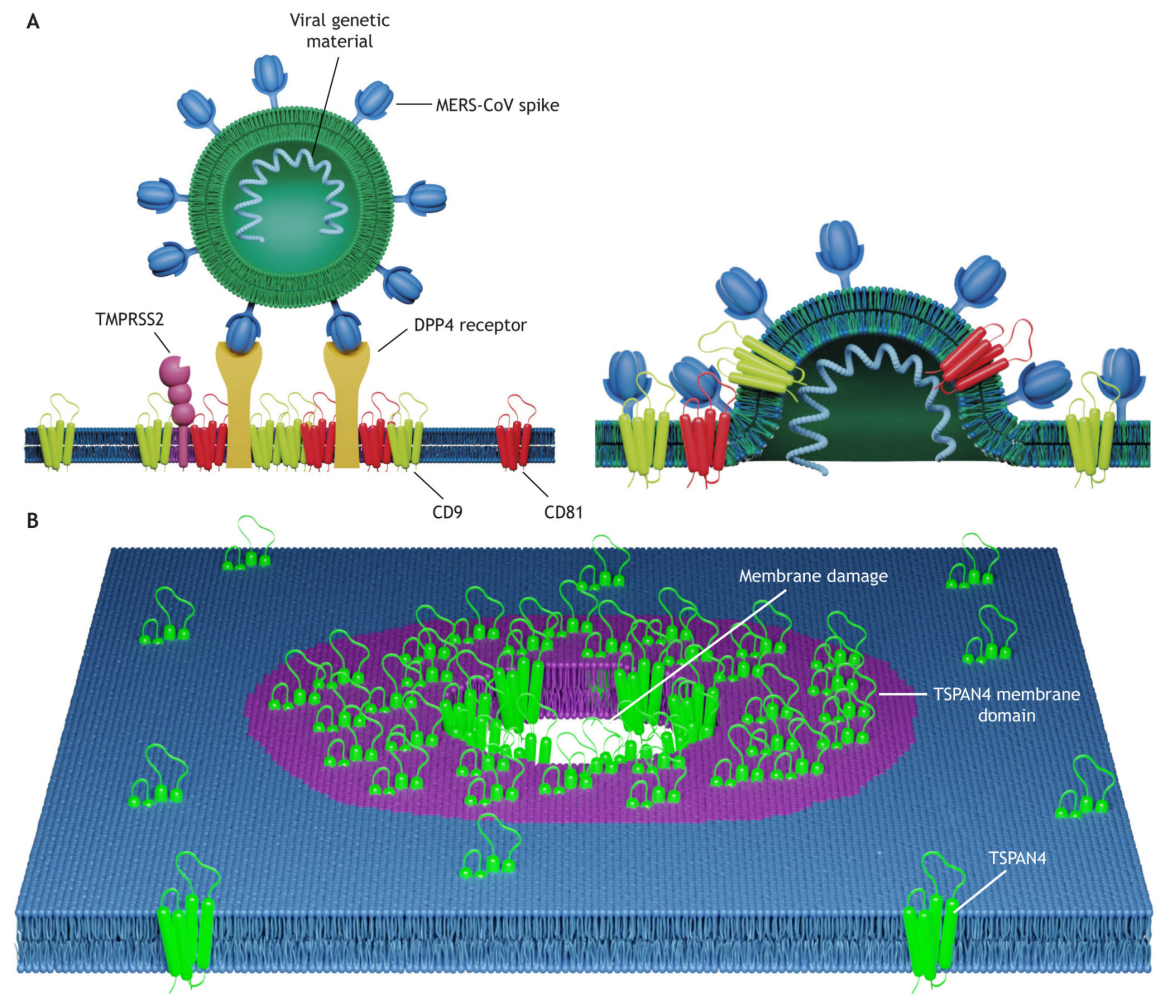
TSPAN membrane domains in fusion and repair. (A) TSPANs promote membrane fusion, as illustrated in this example of MERS-CoV infecting a cell. TSPANs are present on the cell membrane and coalesce receptors (such as DPP4) and proteases (such as TMPRSS2), forming domains (left) that facilitate fusion (right). (B) TSPAN membrane domains facilitate membrane damage repair. Schematic illustration of a TSPAN4 membrane domain (purple) around a membrane hole to aid membrane damage repair.

TEMs are thought to play a role in membrane fusion, where they are suggested to enhance virus–cell fusion of several viruses, including MERS coronavirus (MERS-CoV; Earnest et al., 2017), influenza A (Earnest et al., 2015) and human papillomavirus (Schelhaas et al., 2012). A suggested explanation for such enhancement is that TEMs can selectively coalesce viral and cell receptors, as well as enzymes that cleave and activate viral fusion proteins, thus facilitating virus–cell fusion (Hantak et al., 2019). In further support of this hypothesis, antibodies that bind to TSPANs inhibit infection by coronaviruses and influenza virus. However, when the protease that activates the viral fusion protein is overexpressed in the cell membrane, this inhibition is surpassed, as sufficient protease is available even in the absence of TSPAN-mediated enrichment (Earnest et al., 2015). Thus, viruses exploit TEMs for proper co-engagement with cell receptors, viral fusion proteins and proteases (Fig. 4A).

TSPANs also play a role in cell–cell fusion. It has been proposed that CD9 clusters, which can be induced by the K41 CD9-specific antibody, selectively regulate virus-induced cell–cell fusion. During measles virus infection, these CD9 clusters have been observed to contain virus envelope proteins that promote cell–cell fusion. Conversely, during canine distemper virus infection, CD9 clusters displace viral proteins at cell contact areas, thereby inhibiting fusion (Singethan et al., 2008). Likewise, TSPANs are crucial in the membrane fusion of egg and sperm cells, as knockout of CD9 or CD81 results in infertility in mice (Rubinstein et al., 2006; Jégou et al., 2011). In this fusion process, TSPANs play a dual role: they generate curvature to form a curved microvillus structure while also acting as molecular organizers that might promote protein interactions between the egg and sperm.

In other circumstances, however, CD9 and CD81 can prevent fusion. For example, inhibition of the TSPANs CD9 and CD81 by antibodies or TSPAN knockout significantly increases the fusion of mononuclear phagocytes and myoblasts (Takeda et al., 2003; Charrin et al., 2013). Furthermore, TSPAN8 prevents the docking and fusion of secretory mucin granules to the plasma membrane of goblet cells by modulating the availability of syntaxin proteins that are required for mucin and insulin secretion (Wojnacki et al., 2023). These results suggest that the role of TSPANs in cell–cell fusion varies according to the cell type.

Aside from their role in membrane fusion, it has recently been proposed that TEMs have a functional role in membrane damage repair; TSPAN4 and other TSPANs, like TSP-15 found in *Caenorhabditis elegans*, contribute to the mitigation of cell membrane damage by localizing to the damage site and initiating domain formation (Fig. 4B). These domains are referred to as ‘macrodomains’, and their size is on the micrometer scale. The domains form a ring-like structure around the damage site and act as a physical barrier, limiting the spread of damage and facilitating membrane repair (Huang et al., 2022; Wang et al., 2022). TSPAN4 recruitment to the damage site is associated with the endosomal sorting complexes required for transport (ESCRT) machinery, which likely participates in the damage repair (Huang et al., 2022). In another study, TSP-15 has been found to be recruited to the damage site by the ESCRT machinery, where it interacts with the t-SNARE protein syntaxin-2, which facilitates membrane repair (Wang et al., 2022). In both studies, TSPANs act indirectly in repairing the damage and primarily function as a physical barrier capable of recruiting other repair machinery factors.

## TEMs in cancer

TEMs are also considered to be therapeutic targets as they have a key role in cancerous tumor development (Hemler, 2014). For example, CD37, a leukocyte surface antigen that is prevalent on both healthy and malignant mature B cells (Van Spriel et al., 2012; Barrena et al., 2005), serves as a docking point for monoclonal antibodies used in immunotherapy (Deckert et al., 2013; Bobrowicz et al., 2020). Recent studies have revealed roles of CD37 beyond acting as a mere antibody-binding site, suggesting that therapies aimed at CD37 could offer extra benefits, particularly for patients with recurring or hard-to-treat conditions (Bobrowicz et al., 2020). Studies have demonstrated that T cells expressing a chimeric antigen receptor targeting CD37 are effectively redirected and capable of suppressing the progression of B cell lymphoma tumors (Scarfò et al., 2018; Köksal et al., 2019). Furthermore, CD37 surface expression in B cells predicts significantly better clinical outcomes upon treatment in cases of diffuse large B cell lymphoma (Xu-Monette et al., 2016).

CD82 is another TSPAN that has been implicated in cancer, where it is considered to be a suppressor of tumor metastasis by virtue of its role in regulating cell surface signaling (Miranti, 2009; Tsai and Weissman, 2011), endocytic trafficking (Odintsova et al., 2013), canonical signaling by the Wnt family of secreted glycolipoproteins (Chigita et al., 2012), cell adhesion (Abe et al., 2008) and migration (Ordas et al., 2021). Contrarily, TSPAN7 promotes osteosarcoma metastasis by interacting with β1 integrin (ITGB1) (Shao et al., 2022) and is a promising biomarker for several cancers including multiple myeloma (Cheong et al., 2015), clear-cell renal cell carcinoma (Wuttig et al., 2012), leiomyosarcoma (Davidson et al., 2014) and desmoplastic small-round-cell tumors (Ito et al., 2003). Additionally, CD81 expression in melanoma has been shown to promote tumor growth and metastasis in humans (Hong et al., 2014), and CD81 knockout in osteosarcoma and breast cancer cells attenuates tumor progression and dissemination (Kagiali et al., 2019; Mizoshiri et al., 2019). Mice deficient in CD81 show a decrease in lung metastases when injected with mouse breast cancer tumors (Vences-Catalán et al., 2015).

Given their implication in multiple cancers, TSPANs have become of interest for therapeutic targeting. Recently, it has been demonstrated that a specific antibody (5A6) against CD81 is capable of killing follicular lymphoma tumor cells while sparing normal lymphocytes (Vences-Catalán et al., 2019). Moreover, 5A6 induces CD81 clustering and inhibits breast cancer cell migration and invasion *in vitro*, and can reduce lung metastasis *in vivo* (Vences-Catalán et al., 2021). This inhibition has been suggested to occur through the indirect interaction of CD81 with junctional adhesion molecule A (JAM-A), a transmembrane component of tight junctions known to interact with different integrins and CD9 (Vences-Catalán et al., 2021). As such, CD9 has also been targeted, and a peptide that binds to CD9 has been suggested to impair TEM formation, resulting in anti-metastatic effects. This CD9-binding peptide has been demonstrated to inhibit cell migration and invasion as well as exosome release and uptake *in vitro*, and it reduces lung metastasis *in vivo* (Suwatthanarak et al., 2023). Notably, the abovementioned roles of TSPANs in tumor development involve TSPAN interactions and TEM formation. Thus, given the multifaceted roles of TSPANs in cancer and tumor development, it is imperative to further explore their intricate interactions and therapeutic potential.

## Methods for characterization of TSPAN organization and function

### Fluorescence microscopy

Fluorescence microscopy (FM) has been used to investigate the localization of labeled TSPANs and explore their cellular functions (Takeda et al., 2003; Singethan et al., 2008; van Niel et al., 2011; Zhang et al., 2014; Vences-Catalán et al., 2021; Colbert et al., 2022; Huang et al., 2022; Wang et al., 2022; Dharan et al., 2023; Wojnacki et al., 2023). In particular, single-molecule FM has been used to study the diffusion of TSPANs in the membrane (Barreiro et al., 2008; Charrin et al., 2009; Yáñez-Mó et al., 2009; Yang et al., 2012), revealing that TSPANs can interact with themselves and other proteins, forming membrane domains that are in permanent exchange with the rest of the membrane (Espenel et al., 2008). Although dynamic behavior is common to TSPANs, it has been shown that the dynamics of CD9, CD81 and CD82 are distinguishable, implying that each has a different function (Fernandez et al., 2021). Furthermore, using high-resolution imaging techniques, several studies have investigated TSPAN interactions and TEM formation. For instance, using structured illumination microscopy, it has been demonstrated that TSPAN4 membrane domains and CHMP4B, a component of the ESCRT machinery, are recruited to membrane damage sites, where they are found to be in close proximity but not colocalized (Huang et al., 2022). Photo-activated localization microscopy (PALM) has been used for counting TSPAN molecules in TEMs and to examine the concentration of TSPANs in migrasomes and retraction fibers (Huang et al., 2019). Stimulated emission depletion (STED) microscopy and direct stochastic optical reconstruction microscopy (dSTORM) measurements have also revealed that TSPANs are organized in nanodomains that can be in close proximity but not within the same domain, challenging the TEM notion (Zuidscherwoude et al., 2015; Dahmane et al., 2019).

### Atomic force microscopy

Atomic force microscopy (AFM) can be used to measure the stiffness of membranes and has been applied to membranes containing TSPANs. Nanoindentation measurements of liposomes containing TSPANs suggest that TSPANs increase membrane rigidity (Huang et al., 2019). Another study has used AFM to pull membrane tubes from cells, showing that the tether force increases in the presence of TSPANs (Ordas et al., 2021), which can result from changes in either membrane bending rigidity or membrane tension. Although it is likely that TSPANs can increase membrane bending rigidity, more detailed assays are required to examine the effects of TSPANs on the mechanical properties of natural membranes. For example, conducting nanoindentation measurements on membranes containing TEMs while simultaneously visualizing the membrane to distinguish between TSPAN-enriched and TSPAN-depleted areas would provide valuable insights. This would add complementary information to measurements of averaged mechanical properties of liposomes containing TSPANs (Huang et al., 2019).

### Micropipette aspiration combined with optical tweezers

A combination of micropipette aspiration and optical tweezers was originally used with giant unilamellar vesicles to demonstrate the sensitivity of certain proteins to membrane curvature (Sorre et al., 2012; Aimon et al., 2014). The instrumentation consists of a micromanipulator holding a micropipette and a pressure controller (via hydrostatic pressure or a pump) integrated into a microscope that combines optical trapping and FM (Dimova and Marques, 2020). In this method, a membrane tube is pulled from an aspirated membrane vesicle. The vesicle is considered as flat compared to the membrane tube, which has a diameter in the range of 20–100 nm (Dimova and Marques, 2020). By setting the aspiration pressure, one can regulate the membrane tension of the vesicle and, as a result, the diameter of the membrane tube. This approach has very recently been applied to natural membrane vesicles containing TSPAN proteins, revealing curvature sensitivity of the proteins (Dharan et al., 2022). Micropipette aspiration combined with optical tweezers has also been used to generate membrane swellings following a membrane tension jump obtained by an abrupt increase in the aspiration pressure, providing insight into the process of migrasome formation (Dharan et al., 2023). Beside the complexity of the setup, the main disadvantage of this approach is that the membrane curvature is coupled to the membrane tension. In order to examine these biophysical properties separately, other assays need to be developed.

## Conclusions and future directions

TSPAN transmembrane proteins are involved in various membrane remodeling processes. Membrane shaping and remodeling is central to many physiological processes and has also been implicated in various pathological conditions, such as infertility, infection and cancer. Although the exact mechanism of action of TSPANs in most cellular processes is still unknown, several recent studies have demonstrated that TSPAN functions are associated with their curvature sensitivity, protein enrichment and membrane domain formation. The role of other physico-chemical properties, like membrane tension, needs to be further examined to comprehensively characterize TSPAN functions. In current methods such as tether pulling, membrane tension is coupled with membrane curvature, underscoring the need for innovative assays that can isolate the effects of tension. Additional open questions remain regarding the identity of the associating partners that lead to TSPAN migration from high-curvature membranes to low-curvature membranes, and whether various higher-order structures have different mechanical properties that affect membrane remodeling. Elucidating the exact role of TSPAN proteins in membrane remodeling could lead to better diagnosis and treatment of related diseases.
